# Serum PYCARD may become a new diagnostic marker for rheumatoid arthritis patients

**DOI:** 10.1186/s40001-024-01813-8

**Published:** 2024-04-04

**Authors:** Xue-Li Geng, Yong-Sen Jiang, Chun-Nan Zhao, Ze-Zhi Zhang, Yan-Ling Liu, Pei-Jian Ding

**Affiliations:** 1 Department of Clinical Laboratory, The Affiliated Hospital of Chengde Medical University, No. 36 of Nanyingzi Street, Shuangqiao District, Chengde, 067000 Hebei China; 2 Department of Rheumatology and Immunology, The Affiliated Hospital of Chengde Medical University, Chengde, 067000 China; 3 Department of Gastrointestinal Surgery, The Affiliated Hospital of Chengde Medical University, Chengde, 067000 China

**Keywords:** IL-38, IL-6, PYCARD, Rheumatoid arthritis (RA)

## Abstract

**Background:**

The objective of this investigation is to analyze the levels and clinical relevance of serum PYCARD (Pyrin and CARD domain-containing protein, commonly known as ASC—apoptosis-associated speck-like protein containing a caspase activation and recruitment domain), interleukin-38 (IL-38), and interleukin-6 (IL-6) in individuals afflicted with rheumatoid arthritis (RA).

**Methods:**

Our study comprised 88 individuals diagnosed with RA who sought medical attention at the Affiliated Hospital of Chengde Medical University during the period spanning November 2021 to June 2023, constituting the test group. Additionally, a control group of 88 individuals who underwent health assessments at the same hospital during the aforementioned timeframe was included for comparative purposes. The study involved the assessment of IL-38, IL-6, PYCARD, anti-cyclic citrullinated peptide antibody (anti-CCP), and erythrocyte sedimentation rate (ESR) levels in both groups. The research aimed to explore the correlations and diagnostic efficacy of these markers, employing pertinent statistical analyses for comprehensive evaluation.

**Results:**

The test group had higher expression levels of PYCARD, IL-6, and IL-38 than the control group (*P* < 0.05). Based on the correlation analysis, there was a strong relationship between PYCARD and IL-38 (*P* < 0.01). The receiver operating characteristic (ROC) curve analysis revealed area under the curve (AUC) values of 0.97, 0.96, and 0.96 when using combinations of PYCARD and anti-CCP, IL-38 and anti-CCP, and IL-6 and anti-CCP for predicting RA, respectively. Importantly, all three of these pairs demonstrated superior AUC values compared to PYCARD, IL-38, IL-6, ESR, or anti-CCP used as standalone diagnostic indicators.

**Conclusion:**

PYCARD, IL-6, and IL-38 exhibit promising potential as novel diagnostic markers and may constitute valuable tools for supporting the diagnosis of RA.

## Background

Rheumatoid arthritis (RA) is a chronic autoimmune disease that can cause severe joint injuries, disability, and death [1]. The global average incidence rate of RA ranges between 0.5⁠–1.0% [2]. RA can occur at any age but is most common in people ranging in age between 40–70 years [3]. The incidence of RA is notably higher among women compared to men. Despite ongoing research, the precise etiology of RA remains elusive and is likely multifactorial. Emerging evidence suggests that immune cells and cytokines play pivotal roles in the pathogenesis of RA. Presently, the available serum markers for RA diagnosis are deemed insufficient for accurate and reliable detection. In situations involving RA where significant symptoms are not prominently evident, there exists a substantial risk of missed diagnoses or diagnostic inaccuracies when relying solely on these markers. It is noteworthy that despite the commendable specificity and sensitivity of anti-cyclic citrullinated peptide (anti-CCP), approximately 20–30% of patients with RA test negative for anti-CCP. This underscores the limitations of current diagnostic approaches in capturing the full spectrum of RA cases.

PYCARD serves as an adaptor protein with pivotal roles in assembling inflammatory responses and regulating cell apoptosis. Its functionality as an adaptor molecule is attributed to its structural domains, including the pyrin domain and the caspase activation and recruitment domain (CARD). PYCARD plays a fundamental role in the detection, modulation, and advancement of diverse inflammatory diseases [4]. IL-38 is a member of the IL-1 family that targets autophagy to regulate the proliferation, migration, and invasion of synovial cells in patients with RA [5]. Among inflammatory cytokines, IL-6 is the most prevalent in the synovium of patients with RA and has a strong predictive ability for the severity and progression of RA [6]. It is regarded as a possible biomarker for therapeutic medication response in patients with RA [7]. Drawing from a comprehensive literature review, it was ascertained that PYCARD, IL-38, and IL-6 are implicated in the onset and progression of RA. The primary objective of this study is to assess the expression levels of serum PYCARD, IL-38, and IL-6 in patients with RA and evaluate their diagnostic utility in the context of RA diagnosis.

## Methods

### General data

Between November 2021 and June 2023, a total of 88 patients with RA undergoing treatment in our hospital were included and categorized as the test group. Patients with RA meet the American College of Rheumatology’s 1987 *Diagnostic Criteria for Rheumatoid Arthritis* [8]. Their average age was 53 years and there were 14 males and 74 females. The control group consisted of 88 patients undergoing physical check-ups in our hospital during the same period. Their average age was 53 years and there were 14 males and 74 females.

Inclusion exclusion criteria for the experimental group: (1) All the participants in the study were over the age of 18. (2) Patients with other diseases in the joints were excluded. (3) In addition to RA, patients with a severe dysfunction in the liver or kidneys or a severe metabolic disease, such as thyroid and parathyroid diseases, were excluded. (4) Patients suffering from another severe cardiovascular or cerebrovascular disease, or chronic kidney disease were excluded, along with those having RA. (5) In addition to patients with RA, patients suffering from tumors or tumor metastasis to bones were excluded. (6) Patients taking anti-cancer medications or other drugs affecting bone metabolism were excluded. (7) Women who were pregnant or lactating were excluded. (8) Patients with other autoimmune diseases were excluded.

Inclusion criteria for the control group: (1) All study subjects were older than 18 years old; (2) no joint diseases; (3) no metabolic diseases (4) no cardiovascular or cerebrovascular diseases or chronic kidney diseases; (5) no tumors and bone metastases. Exclusion criteria for the control group (1) recent drug use (2) pregnant or lactating women; (3) autoimmune diseases.

This study was conducted with approval from the Ethics Committee of The Affiliated Hospital of Chengde Medical College. This study was conducted in accordance with the declaration of Helsinki. Written informed consent was obtained from all participants. Research ethics approval number: CYFYLL2020250.

### Research methods

General clinical data of all the study participants were collected, including gender, age, disease duration, and others. Upon waking up, 4 mL of fasting blood specimen was collected from all the study participants, which was then placed in a vacuum tube without anticoagulant. The specimens were placed at room temperature for half an hour for natural clotting, then centrifuged at a speed of 4000 r/min for 15 min. A volume of 500 μL of the supernatant was extracted and subsequently transferred into an EP tube, where it was preserved in an ultra-low temperature freezer at − 80 °C. Serum samples from all study participants were collectively subjected to analysis. The quantification of PYCARD, IL-38, and IL-6 was carried out utilizing the enzyme-linked immunosorbent assay (ELISA) technique, which was provided by Wuhan Jining Biotechnology. Additionally, the measurement of anti-CCP levels was conducted using the electrochemiluminescence method, supplied by Roche. The samples were analyzed using the enzyme immunoassay analyzer BIO-RAD680. The erythrocyte sedimentation rate (ESR) was measured using the appropriate tools. Minimum detection limits: IL-38 minimum detection limits 25 pg/mg; PYCARD minimum detection limits 78.125 pg/mg; IL-6 minimum detection limits 0.6 ng/mg.

### Statistical analysis

Data analysis was performed using the SPSS26.0 software. Non-normally distributed data are expressed as interquartile range (IQR), while normally distributed data are expressed as mean ± standard deviation. If the data of the two groups were normally distributed, the independent samples t-test were used to test the data. If the data of the two groups were not normally distributed, they were tested using the Mann–Whitney U test. The ROC curve, AUC, and Youden’s index were measured.

## Results

### Baseline characteristics and expression levels in test and control groups

Using the Mann–Whitney U test. There were statistically significant differences in the expression levels of PYCARD, IL-38, IL-6, ESR, and anti-CCP between the test group and the control group (*P* < 0.01) (Table 1).

**Table 1:** Demographic data about the two groups

	Group	M (P25, P75)	Median difference (95%CI)	Z	P
IL-38 (pg/ml)	Test group (n = 88)	559.36 (399.29, 779.18)	380.7 (310.51, 434.05)	8.14	P < 0.01
	Control (n = 88)	135.67 (112.97, 258.89)			
PYCARD (pg/ml)	Test group (n = 88)	1968.07 (1569.06, 2597.26)	0.32.94 (862.18, 1236.505)	8.83	P < 0.01
	Control (n = 88)	871.82 (739.15, 1134.18)			
IL-6 (ng/ml)	Test group (n = 88)	3.58 (2.53, 4.98)	1.78 (1.39, 2.19)	7.50	P < 0.01
	Control (n = 88)	1.62 (1.31, 2.13)			
ESR (mm/h)	Test group (n = 88)	22.5 (13, 37.5)	14 (11, 18)	8.82	P < 0.01
	Control (n = 88)	8 (7, 10)			
Anti-CCP (u/ml)	Test group (n = 88)	228 (25.22, 499.55)	219.1 (164.9, 300.6)	7.42	P < 0.01
	Control (n = 88)	8.4 (7.8, 9.3)			

### Correlation analysis of PYCARD, IL-38, IL-6, ESR, anti-CCP, and DAS28 levels

Based on the results of the analysis, there was a significant correlation between PYCARD and IL-38, between PYCARD and DAS28, between PYCARD and ESR, and between PYCARD and IL-6, with the correlation coefficients being 0.76, 0.64, 0.42, and 0.41 for each pair, respectively. (Table 2).

**Table 2:** Correlation analysis between PYCARD, IL-38, IL-6, ESR, anti-CCP, and DAS28

Items	IL-38	IL-6	PYCARD	ESR		DAS28
IL-38	1					
IL-6	0.29	1				
PYCARD	0.76	0.41	1			
ESR	0.32	0.16	0.42	1		
anti-CCP	0.06	0.11	-0.07	0.11	1	
DAS28	0.05	0.03	0.64	0.27	0.16	1

### Diagnostic significance of serum PYCARD, IL-38, IL-6, ESR, and anti-CCP levels in RA

As indicated by the ROC curve, the AUC was 0.97 when serum PYCARD and the anti-CCP levels were jointly used to predict RA. This value was greater than the AUC of IL-38 and the anti-CCP level in combination and IL-6 and the anti-CCP level. In addition, the AUC values of the above three combinations were greater than those of PYCARD, IL-38, IL-6, ESR, and anti-CCP alone in predicting RA (Table 3).

**Table 3:** Significance of serum PYCARD, IL-38, IL-6, ESR, and anti-CCP levels in predicting RA

Factor	AUC	95%CI	Optimal cut-off value	Sensitivity (%)	Specificity (%)	Youden’s index
IL-38	0.85	(0.7966, 0.9139)	298.27	84.1	80.7	0.648
IL-6	0.82	(0.7602, 0.894)	2.25	81.8	77.3	0.591
PYCARD	0.89	(0.835, 0.9354)	1291.6	85.2	81.8	0.67
ESR	0.89	(0.8294, 0.941)	12.5	78.4	92	0.704
anti-CCP	0.82	(0.7471, 0.9003)	12.5	79.5	98.9	0.784
IL-38/anti-CCP	0.96			84.1	94.3	0.784
IL-6/anti-CCP	0.96			98.9	89.8	0.887
PYCARD/anti-CCP	0.97			96.6	87.5	0.841

## Discussion

IL-38, a constituent of the IL-1 cytokine family, was initially designated as IL-1HY 2 in 2001. In the human genome, the IL-38 gene is situated on chromosome 2 q13-14.1. Emerging from extensive research endeavors, a discernible association has been elucidated between IL-38 and inflammatory disorders, notably exemplified by its relevance in the context of RA [9]. We assessed the potential of IL-38 to serve as a biomarker of RA and discovered that the concentration of serum IL-38 was significantly higher in the test group than in the control group (559.36 pg/mL vs 135.67 pg/mL). Based on the correlation analysis, the correlation coefficient between IL-38 and PYCARD was 0.76. The diagnostic analysis determined that the predictive sensitivity and specificity of IL-38 were 84.1 and 80.7, respectively. According to the analysis of combined prediction, IL-38 and anti-CCP in combination had a predictive sensitivity and specificity of 84.1 and 94.3, respectively, with the AUC being 0.96. A combination of IL-38 with other indicators had a high predictive specificity. IL-38 is capable of enhancing the specificity in the diagnosis of RA and has the potential to improve the diagnostic specificity of RA.

IL-6 is a secreted protein with a molecular weight of 26 kDa. Mature IL-6 contains 184 amino acids, 2 *N*-glycosylation sites, and 4 cysteine residues. Bone marrow cells such as macrophages and dendritic cells rapidly produce IL-6 at the sites of tissue injuries upon recognition of sterile or non-sterile pathogens by toll-like receptors (TLRs) [10]. IL-6 is a multifunctional cytokine that participates in the acute response of autoimmune and inflammatory diseases, including autoimmune hemolytic anemia, systemic sclerosis, inflammatory bowel disease, and rheumatoid arthritis, among others [11]. Elham et al. concluded that the level of serum IL-6 is positively correlated with the severity of RA [12, 13]. We assessed the potential of IL-6 to serve as a biomarker of RA and discovered that the level of serum IL-6 in the test group was significantly higher than in the control group (3.58 pg/mL vs 1.62 pg/mL). Correlation analysis unveiled a correlation coefficient of 0.41 between IL-6 and PYCARD. In terms of diagnostic utility, IL-6 exhibited a sensitivity of 81.8% and a specificity of 77.3% in predicting and analyzing RA.

Furthermore, when IL-6 was employed in combination with anti-CCP for predictive and analytical purposes in RA, a notably heightened sensitivity of 98.9% and specificity of 89.8% were achieved. These findings were underpinned by an impressive area under the curve (AUC) value of 0.96, indicating the robust diagnostic potential of the IL-6 and anti-CCP combination in RA assessment. The predictive specificity of a combination of IL-6 and other markers is quite high. IL-6 is capable of enhancing the diagnostic specificity of RA and has the potential to improve the diagnostic specificity of RA.

PYCARD is a known adaptor protein that connects the activated inflammasome sensor to the caspase-1 precursor effector molecule. It promotes the self-catalytic activation of caspase-1 by recruiting caspase-1 precursor [14]. The primary function of caspase-1 is to convert inactive interleukin precursors into active interleukins [15]. Within the scope of this investigation, we observed a substantial disparity in serum PYCARD concentrations between the test and control groups, with levels measuring 1968.07 pg/mL and 871.82 pg/mL, respectively. Furthermore, our analysis revealed notable correlations between PYCARD and other parameters, including IL-38, IL-6, and ESR, characterized by correlation coefficients of 0.76, 0.42, and 0.41, respectively. The sensitivity and specificity of PYCARD in predicting and analyzing RA were 85.2 and 81.8, respectively, according to the diagnostic analysis. In the context of predicting and analyzing RA, the combination of PYCARD and anti-CCP exhibited a notably high sensitivity of 96.6% and a specificity of 87.5%, with an exceptional AUC value of 0.97—the highest among all considered markers. Based on the compelling findings of this study, we assert that PYCARD holds promise as a novel serum marker for enhancing the diagnostic accuracy of RA.

To sum up, the expression of PYCARD, IL-38, and IL-6 was significantly elevated in patients with rheumatoid arthritis. The correlation analysis of PYCARD with IL-38, IL-6, ESR, anti-CCP, and DAS28 showed that PYCAR was correlated with the disease severity of patients with rheumatoid arthritis as well as other inflammatory factors, and played an important role in rheumatoid arthritis. Upon evaluation using ROC curves, PYCARD emerged as the most sensitive marker, while anti-CCP demonstrated the highest specificity in predicting RA, with sensitivity and specificity values of 85.2% and 98.9%, respectively. These findings collectively underscore the diagnostic potential of PYCARD and anti-CCP, affirming their significance in RA diagnosis. Although IL-6 and anti-CCP in combination had the highest predictive sensitivity (98.9) and IL-38 and anti-CCP in combination had the highest predictive specificity (94.3), PYCARD and anti-CCP in combination had the highest AUC in predicting RA of 0.97. PYCARD provides new evidence for the diagnosis of RA. Given the limited sample size in this study, future research endeavors should prioritize the inclusion of larger sample sizes to bolster the robustness and reliability of the findings. Moreover, for a more comprehensive investigation into the role of PYCARD, forthcoming studies should aim to augment the assessment of inflammatory factors within peripheral serum. Additionally, it is imperative to subject the outcomes of this study to further validation and scrutiny to enhance their credibility and generalizability.

## Conclusion

The current body of research strongly suggests that PYCARD exhibits elevated expression levels in individuals afflicted with RA and holds the potential to augment the diagnostic efficacy of RA. The analyses and findings presented in this study underscore the robust association of the inflammatory factor PYCARD with RA, hinting at its potential role as a principal regulator of other inflammatory factors such as IL-38 and IL-6. Further investigations focused on PYCARD may unveil the underlying pathogenic mechanisms of RA, with far-reaching implications for the advancement of diagnostic, therapeutic, and prognostic strategies to benefit patients with RA more comprehensively.

## Data Availability

The data that support the findings of this study are available from the corresponding author, upon reasonable request.

## Abbreviations

PYCARD: Pyrin and CARD domain-containing protein, commonly known as ASC—apoptosis-associated speck-like protein containing a caspase activation and recruitment domain
IL-38: Interleukin-38
IL-6: Interleukin-6
RA: Rheumatoid arthritis
anti-CCP: Anti-cyclic citrullinated peptide antibody
ESR: Erythrocyte sedimentation rate
ROC: Receiver operating characteristic
AUC: Area under the curve
CARD: Caspase activation and recruitment domain
ELISA: Enzyme-linked immunosorbent assay
TLRs: Toll-like receptors
